# The Production and Characteristics of a Mouse's Embryonic Stem Cell Lineage, Transfected by the Glia Neurotrophic Factor and Gene Fused with the Green Fluorescent Protein Gene 

**Published:** 2009-04

**Authors:** E. L. Arsenieva, I. V. Kuzmin, E. S. Manuilova, E. V. Novosadova, E. V. Murkin, G. V. Pavlova, V. Z. Tarantul, I. A. Grivennikov

**Affiliations:** 1 Molecular Genetics Institute, Russian Academy of Sciences, Moscow;; 2 Gene Biology Institute, Russian Academy of Sciences, Moscow

## Abstract

The influence that the expression of the human (glial-derived neurotrophic factor (GDNF)) neurotrophic factor has on the morphology and proliferative activity of embryonic stem cells (SC) of a mouse with R1 lineage, as well as their ability to form embroid bodies (EB), has been studied. Before that, using a PCR (polymerase chain reaction) coupled with reverse transcription, it was shown that, in this very lineage of the embryonic SC, the expression of the receptors' genes is being fulfilled for the neurotropfic RET and GFRα1 glia factor. The mouse's embryonic SC lineage has been obtained, transfected by the human GDNF gene, and has been fused with the "green" fluorescent protein (GFP) gene. The presence of the expression of the human GDNF gene in the cells was shown by northern hybridization and the synthesis of its albuminous product by immunocitochemical coloration with the use of specific antibodies. The reliable slowing-down of the embriod-body formation by the embryonic SC transfected by the GDNF gene has been shown. No significant influence of the expression of the GDNF gene on the morphology and the proliferative activity of the transfected embryonic SCs has been found when compared with the control ones.

## INTRODUCTION

Glial-derived neurotrophic factor (GDNF) belongs to the GDNF-similar ligand (GFL) family, which includes neurturin (NRTN), persipin (PSPN), and artemin (ARTN). All of these are necessary for the survival of the dopaminergic neurons of the mesencephalon, as well as for peripheral sensory and sympathetic neurons (with the exception of PSPN) [12, 14]. In addition, beyond the nervous system, GDNF fulfills the following important functions: it regulates the differentiation of spermatogonia and is needed for the embryonic development of kidneys [14]. 

All representatives of the GFL family influence the cells through bonding with the heteroreceptor complex, which contains the RET receptor and GFRα co-receptor [14]. The specificity of the RET receptor activation, in relation to different factors of the GFL family, depends on the GFRα type contained in the cells. GDNF, NRTN, ARTN, and PSPN have strong affinities to GFRα1, GFRα2, GFRα3, and GFRα4 co-receptors, respectively. GFL binding with the "alien" GFRα is possible, but it happens with less efficiency. It has been shown that GDNF increases the survival rate of the dopaminergic neurons of rats' embryonic mesencephalon in a specific way, making the dopamine metabolism stronger; it also increases the differentiation level of tyrosine hydroxilase-positive cells (TH+), raising the axons' growth and increasing the dimensions of the cell's body. Analogous effects have also been observed in rats' mesencephalon dopaminergic neurons [12]. Similar results have been obtained on fruit-fly transgenic lines. It appeared that a distinct synthesis of tyrosine hydroxilase has been found in the fruit fly's lines bearing the GDNF gene and in differentiating nerve cells, and the fruit fly's cells bearing the GDNF gene were actively producing acetylcholinesterase [2, 3] 

Embryonic SCs are able to self-renew and differentiate to the derivatives of all three primary embryonic leaves both in vitro and in vivo. Most importantly, in the course of the embryonic SCs, the differentiation of the order of expression and the tissue-specific genes correspond to the order of these processes during the development of the organism in vivo [7]. 

The ability of the SCs to differentiate in definite directions in vitro makes it possible to study the cell and the molecular mechanisms of early development on this type of model, as well as to receive different types of donor cells for transplantation. Prioritized embryonic SC differentiation in a definite direction is achieved only with specific cultivating conditions [9] after exogenous growth factors are applied and after differentiation [7] by way of their genetic modification [1, 10, 11, 13]. The first research works on embryonic SC-directed differentiation were conducted mostly on mouse embryonic SCs, whose differentiation with the help of the above-mentioned methods could be directed to the hemopoetic cells [16], cardiomyocytes [11], insulin-secreting cells [15], and neurons and glia cells [4, 6]. The use of the combined methods of genetic manipulation with mouse embryonic-SC cultivaton on the feeder layer of stromal cells has shown a significant increase in the number of dopaminergic neurons (approximately twice as many) in the pilot test as in the control one. The results of tests on the transplantation of such cells have shown the efficient integration of the TH+ cells into the mouse's striatum [10]. The induction of dopaminergic differentiation was shown on human embryonic SCs with condition of their cultivation on the stromal P06 cells [18]. Preserving the vitality of the dopaminergic neurons during their transplantation remains essential. Buytaert-Hoefen et al. [5] showed that, in order to improve the vitality of the dopaminergic neurons, the presence of a factor such as the GDNF factor or astrocytes from the striatum is necessary. Japanese researchers [17], using the primate embryonic-SC cocultivating technique on Sertoli cells secreting GDNF, found not only an increase in the number of induced dopaminergic neurons over the control-test number, but they also successfully transplanted these cells to the mouse's striatum, which has a pathology similar to Parkinson's disease in humans. Embryonic SCs obtained as a result of definite experimental manipulations, including genetic ones, and possessing a specific type of directed differentiation may be used in the future in cellular therapy of several critical human illnesses.

In connection with this, examining embryonic SCs with increased GDNF expression genes is essential. In order to achieve that, in this article embryonic-SC polyclonal cultures of mice were obtained and transfected with the human GDNF gene fused with the gene that codes the "green" fluorescent protein (GFP); they were then described.

## EXPLORATORY PROCEDURE

CULTIVATING MOUSE EMBRYONIC SCS Cultivating the embryonic SCs was done at 37°C and 5% CO_2_ in α-MEM (Sigma, United States) medium containing 15% cow fetal serum (CFS) (Gibco, United States), 0.1 mM 2- mercaptoethanol, 2 mM L-glutamine, nonessential amino acids (Gibco, United States), nucleosides, vitamins, and gentamicin antibiotic (20 microgram/ml). As a nourishing (feeder) layer for the embryonic Scs, primary fibroblasts received from embryonic mice in the 11th-12th day of development were used, the proliferation of which was blocked by mitomycin C (3 microgram/ml). The medium for growing the primary fibroblast culture was DMEM (Sigma, United States) containing 10% CFS, 2 mM L-glutamine, and gentamicin antibiotic (20 microgram/ml). When cultivating embryonic SCs without the feeder layer, leukemia inhibiting factor (LIF) (Sigma, United States) was added in a final concentration of 10 ng/ml, which blocked the spontaneous differentiation of these cells. The cells were reseeded and medium-changed every 3 days.

Estimating the Expression Level of the RET and GFRα1 Receptors with the Help of Polymerase Chain Reaction Coupled with the Reversed Transcription (RT-PCR) The total DNA from the non-differentiated embryonic Scs, which were cultivated without the feeder layer in the presence of LIF, were extracted following the manufacturer's recommendations by the method of phenol-chloroform extraction with the use of the YellowSolve (Clonogen, United States) kit. The reversed transcription procedure was held with the use of the Silex Company (Russia) kit according to the protocol and recommendations of the manufacturer. kDNA synthesis was done on 2 micrograms of the total RNA for 1 h at 37° C in 25 microliters of reaction mixture containing 0.05 micrograms of accidental hexaprimers and 100 units of MMLV (molonеy murine leukemia virus) reverse transcriptase. After the cessation of the reaction (10 min incubation at 70°C), the samples of kDNA were kept at -20° C.

Polymerase chain reaction (PCR) was conducted in a 25 microliter reaction mixture composed of а Taq-buffer, 1.5 mM of the dNTP mixture, 1.25 units of "colored" Taq polymerase (Sintol Russia), 0.5 micro grams of kDNA sample, and 10 Pmol of each primer. 

The primers' sequences used in the tests are given below. [8]

For the RET gene: 5'-CCTCCGTGACAGCCGCAAGA-3' (forward), 5'-GGGAATCCGGCCCTTGCTTT-3' (reverse); size of the product 297 bps

For the GFRα1 gene: 5'-TCATTGGCAGAAACATCGTAG-3' (forward), 5'-GCTCAGCTTGCTTTACAGTCC-3'(reverse); size of the product 285 bps 

Conditions of the PCR were the same for both fragments: 5 min at 52°C, 5 min at 95°C, then 35 cycles, including at 95°C for 30 sec; at 68°C for 30 sec; at 72°C for 30 sec, and at 72°C for 10 min. The products of the PCR were separated by electrophoresis in 1.5% agarose gel with visualization with the help of lower bromide etidium.

Northern hybridization mRNA was extracted from the embryonic SCs using TRI REAGENT (Sigma, United States) according to the protocol of the manufacturer and separated by electrophoresis in 1.5% agarose gel. Then, mRNA was transferred on the nylon membrane. The probe for GDNF gene was marked by the α-32P АТР with the help of the Random Prime Labeling Kit (Promega, United States) in accordance to the instructions of the manufacturer. Prehybridization was carried out in the hybridization buffer (Х5 SSPE, 0.1% SDS, Х5 Denhardt solution) for 1 h at 65°C. The labeled probe was denaturized for 5 min at 100°C and added to the hybridization buffer.

Hybridization was conducted for 16-20 hours at 65°C on the water bath. Then, the filter was washed 3 times (for 15 min each time) in the Х0.l SSC, 0.1% SDS solution and wrapped in a waterproof plastic film and put into the photo film holder with the photo film. Then it was exposed at -70°C for 1 to 5 days.

**Fig.1.**
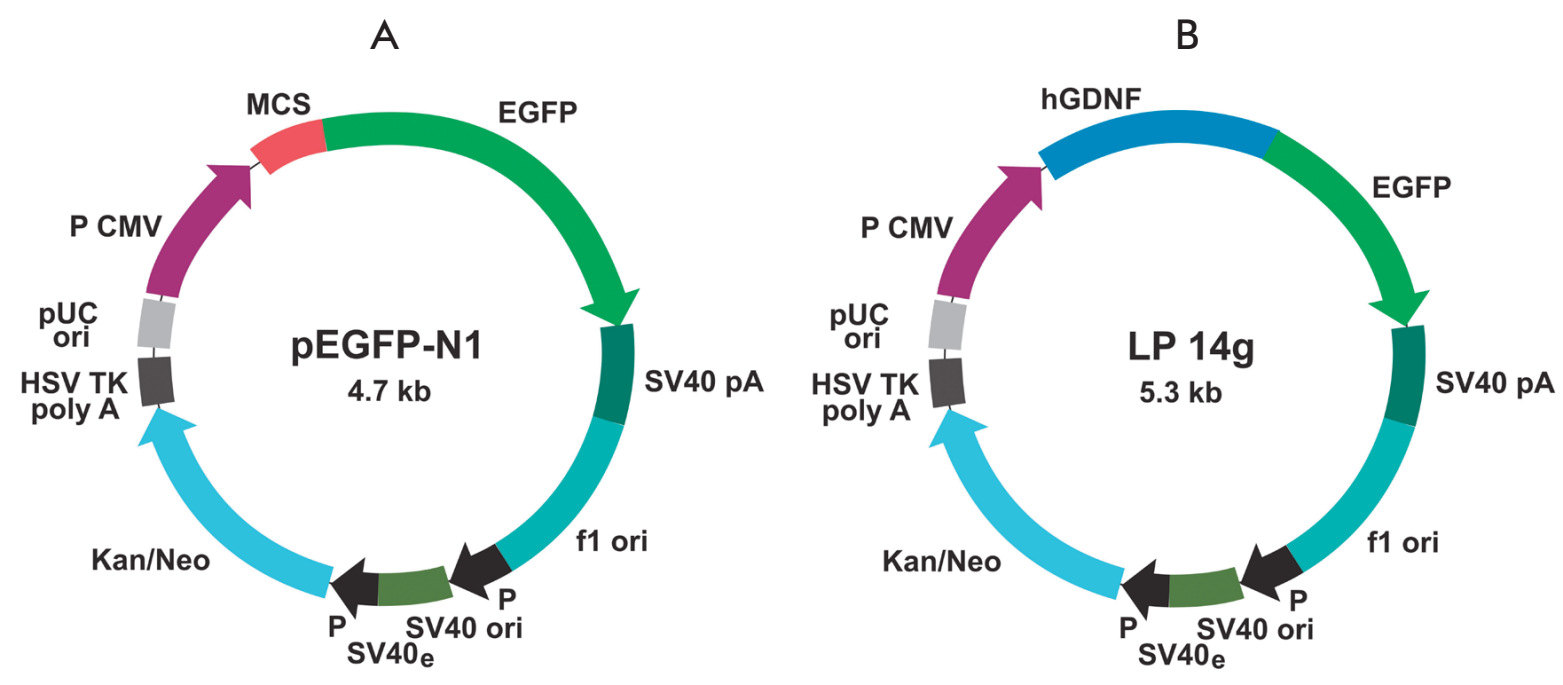
pEGFP-N1 and LP14g plasmid construction schematics for embryonic SCs transfection. Kan/Neo is a gene providing resistance to antibiotics, EGFP is the "green protein" gene, MCS is the sequence containing restriction sites, and hGDNF is the human GDNF gene insertion.

Recombinant plasmids Following genetic engineering, constructions were used for the cells transfection. pEGFP-N1 control plasmid (Fig.1A) contained the GFP gene under the (CMV) cytomegalovirus promoter and a gene resistant to neomycin neo antibiotic. The size of the plasmid is 4.7 thousand bts. LP14g plasmid (Fig.1B) carrying the human GDNF gene fused with the GFP gene was constructed on the pEGFP-N1 basis. The plasmid's size is 5.3 thousand bps, and the plasmid's structures are shown in Fig.1.

Embryonic SCs transfection and selection Insertion of the plasmid DNA into embryonic SCs was carried out with the help of electroporation using a SUM4 device (V.A.Engelgardt Molecular Biology Institute, Russian Academy of Sciences) at the following characteristics selected through experiments: impulse duration was 1.5 microseconds, and 400 V voltage. For the transfection of one million cells, 6-8 micrograms of plasmid DNA were used. Trnsfected cells were seeded, 300,000 in each Petri cup (35 mm diameter, gelatin covered (0.01%) in 2 ml of the standard for the embryonic-SC medium with the addition of LIF (10ng/ml)). Selection began on the second day after seeding by adding the antibiotics G418 (200 microgram/ml) to the medium. The selective medium was changed every 3-4 days. Polyclonal cultures of each transfection version were gathered on the 10th day from the cups in the form of a summary pool of G418-resistent clones. Transfected cells were analyzed visually under an Axioscope (Karl Zeiss, Germany) microscope. The average transfection efficiency was about 10^-4^ .

Determining the proliferative activity of the embryonic SCs An estimation of the proliferative activity of the control and transfected lines was conducted on the third day after seeding by directly calculating the cells under an Olympus CКХ41 (Olympus, Japan) microscope in the Goryayev chamber.

Embroid body production For the induction of differentiation with the production of embroid bodies (EB), the embryonic SCs were isolated from the fibroblasts of the feeder layer. In order to do this, the cells were processed in tripsin, centrifugated, and then the obtained suspension was incubated in Petri cups (d = 60mm) (Nunc, Denmark) for 15-30 min. During that time, most of the mass of fibroblasts sticks to the cup's bottom while the embryonic SCs remain in the suspension. To form EB, the embryonic SCs suspension was transferred either to the Petri cup (d = 60mm) (Nunc, Denmark) in an amount of 500,000 or to the 96-well immunology plate (1,000 cells on each well) and then placed into a CO_2_-incubator (5% CO_2_). Calculating the number of the produced EB was done on the 3th-4th cultivation day. 

To form single EBs, the "hanging drop" method was applied [7]. Embryonic SCs cultivated on the gelatin substrate in the medium with LIF (Sigma, United States) added were used for this. Cells were processed with tripsin, and a suspension with 25,000 cells/ml was prepared. Fifty drops of 20 microliters containing 500 cells each were placed on the lid of a Petri cup (Nunc, Denmark) 60 mm in diameter. In order to get a humid atmosphere, Hanks solutions (2 ml) were added to the cups and then they were placed into the CO_2_-incubator (5% CO_2_). On the third cultivating day, the produced EBs were transferred to the 4-well substrates (the diameter of one well is 15 mm) precoated with gelatin for further differentiation.

An Immunocytochemical Analysis of the Presence of the GDNF Human Gene Protein Product in the Transfected Lines of Embryonic SCs Three-day-old EBs were cultivated in the 4-well tray with a 15-mm well diameter (Nunc, Denmark) (3-4 bodies per one well) processed with 0.01% gelatin in 700 microliters of embryonic SCs cultivating medium not containing LIF. On the 7th cultivation day, differentiated cells were fixed by 4% paraformaldehyde in PBS for 30 min at room temperature. After being washed three times, PBS cells were pre-incubated in the PBS solution containing 0.1% Triton X-100 and 5% FBS for 15 min at room temperature. Chicken polyclonal antibodies were used as primary antibodies against human GDNF (Promega, United States) in a 1 : 30 dilution in the PBS-0.1% Triton-5% FBS solution. Incubation was conducted for one night at +4°C. After washing PBS three times, secondary biotynilated rabbit antibodies were brought on against chicken immunoglobulins (Imtek, Russia) in a dilation of 1 : 500 and incubated for 2 h at room temperature and then processed in a peroxidase conjugate with streptavidin (Imtek, Russia) in a dilution of 1 : 400 for 1 h at room temperature. The reaction was developed by the use of a 0.015% solution of 3-amino-9-ethylcarbasol (Sigma, United States) in 50 mM Tris-citrate рН 7.0 buffer containing 0.06% Н_2_О_2_. Coloring development was visually controlled with using an Olympus CKX41 (Olympus, Japan) microscope. The reaction ended with the cells being washed three times with distilled water.

Statistical analysis Results were processed with the help of Sigma Plot (Jandel Scientific, United States) software. The validity of the group average differences were estimated with the help of dispersion analysis (one-way ANOVA). Results were represented in the form of standard error (mean ± SEM). Values of differences at p less 0.05 were considered valid.

## RESEARCH RESULTS

**Fig.2.**
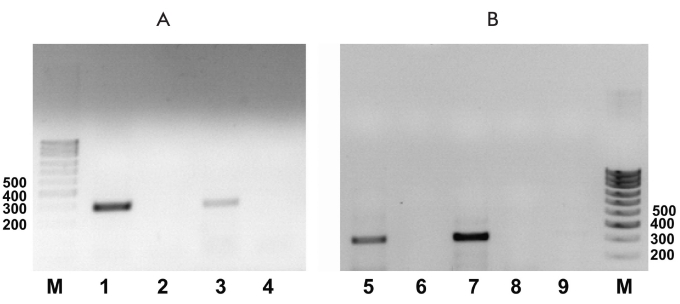
(A) is the GFRα1 receptor's expression. M is the fragments lengths marker, 1 is the mouse's hippocampus (positive control), 2 is the mouse's hippocampus control on the genomic DNA impurity, 3 is the line R1 embryonic SCs, and 4 is the line R1 embryonic SCs control on the genomic DNA impurity. The length of the product is 285 bps (B) is the RET receptor expression, 5 is the hippocampus (positive control), 6 is the mouse's hippocampus control on the genomic DNA impurity, 7 is the R1 line embryonic SC R1- line, 8 is the R1 line embryonic SC control on the genomic DNA impurity, and 9 is the water. The length of the product is 297 bps

During the initial stages of work, the presence of en expression of the receptors for GDNF - RET and GFRα1 on the мРНК level was determined in the embryonic SCs of the initial R1 line mouse with the help of RT (PCR). The findings are presented in Figs. 2A and 2B. It is seen from this information that embryonic SCs are expressed by both GFRα1 and RET, though with different efficiencies.

**Fig.3.**
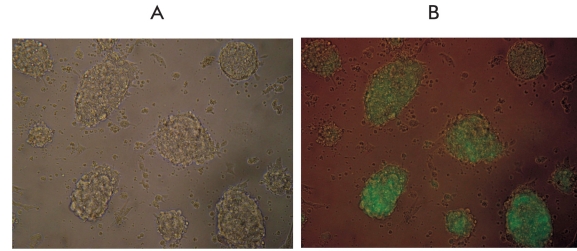
es-GDNF line cells colonies in (A) the light microscope and (B) fluorescent microscope (× 100).

**Fig.4.**
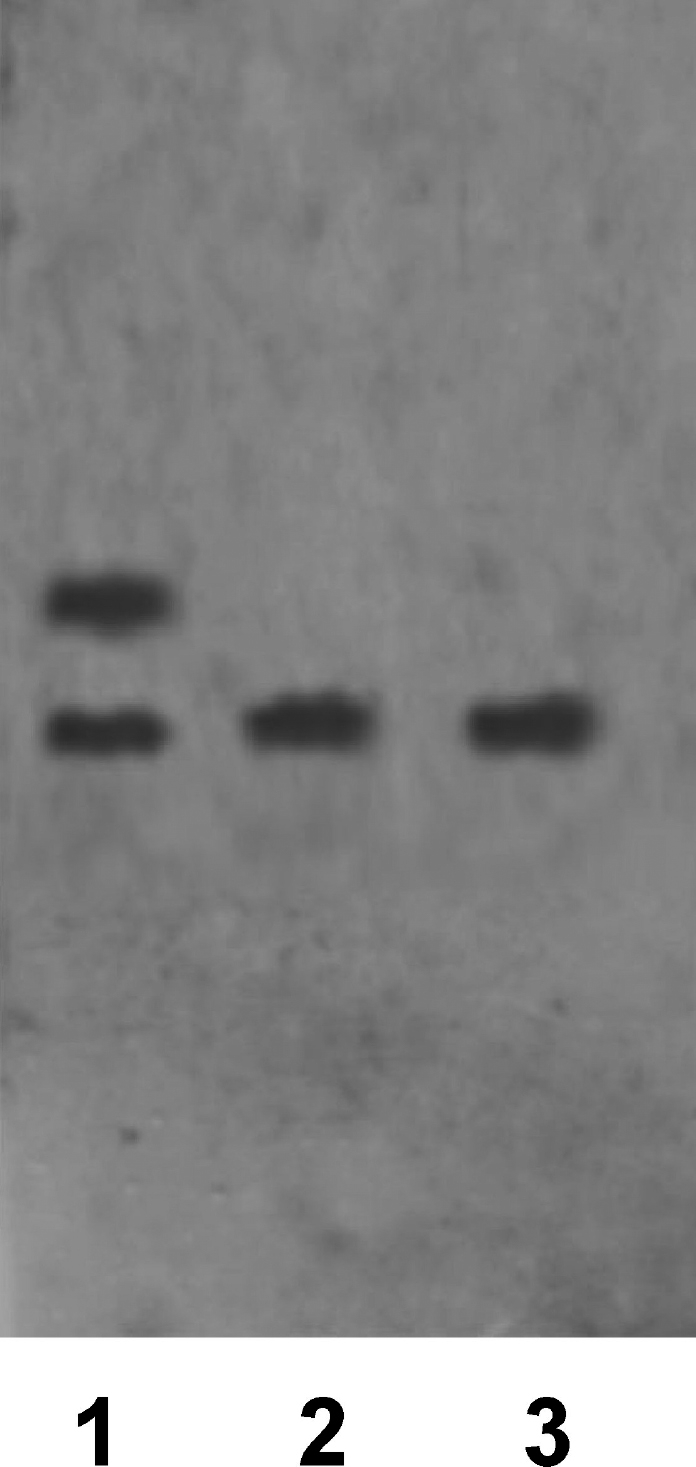
GDNF gene expression analysis in embryonic SCs with the help of northern hybridization. (1) RNA from embryonic SCs transfected with a LP14g plasmid carrying the human GDNF gene fused with a GFP gene. (2) RNA from embryonic SCs transfected with a pEGFP-N1plasmid carrying the GFP gene. (3) RNA from non-transfected embryonic SCs.

**Fig.5.**
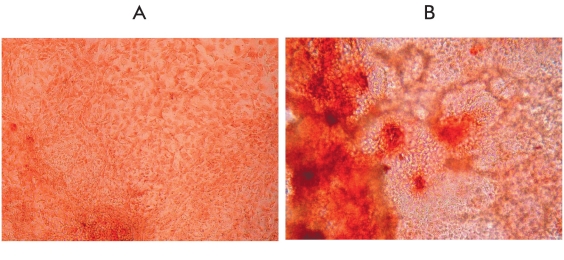
Immunocytochemichal coloring of the es-GFP line cells (A) and es-GDNF (B) by the antibodies to human GDNF (× 100).

Two lines were obtained as a result of transfection with corresponding recombinant plasmids and further selection of the embryonic SCs: es-GDNF (the line bearing the GDNF gene fused with the "green protein" gene) and es-GFP (the control line only, with the "green protein" gene). The presence of the GFP gene insertion makes detecting the cell colonies easier after the transfection has been carried out; it also makes it easier to investigate the ways and patterns of the embryonic SCs differentiation, especially in in vivo tests. The green glow was observed in more than 80% (Fig. 3B) of transfected cells in the received cells' lineages. With the help of northern hybridization, coupled with the use of the labeled GDNF fragment, its expression in the es-GDNF line cells (Fig.4) was shown. Besides, as can also be seen from this picture, the expression of the endogenous GDNF gene happens in the embryonic SCs. To detect the protein product synthesis in this gene, the differentiated cells of the es-GDNF and es-GFP were colorated with the help of polyclonal to human GDNF antibodies. The results of the experiments are presented in Figs. 5 A and 5B. The slight pink coloring (Fig. 5A) is due to the fact that embryonic SCs seemingly synthesize their own GDNF in small amounts. Indirect confirmation of this was, as has already been mentioned, obtained with the help of northern hybridization (Figs. 4 2 and 4 3). Brightly colored cell clusters are evidence of human GDFN synthesis in the es-GDNF line cells (Fig. 5B).

The influence of the human GDNF gene expression on the proliferative activity of the embryonic SCs was studied during the next stage. The proliferative activity of the control and experimental cell comparisons did not detect valid differences between them (this data has not been cited). These results allow us to suggest that the human GDNF gene product does not play an essential part in the cell-cycle regulation of these cells. 

**Fig.6.**
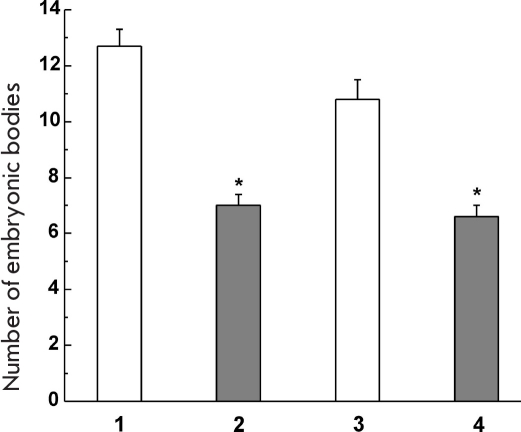
hGDNF gene expression influence on the formation of the embroid bodies transfected by embryonic SCs in vitro. (1, 2) number of formed EBs on the 3rd day after cell seeding. (3, 4) number of formed EBs on the 4th day after cell seeding. White columns indicate the (es-GFP) control. Grey columns are the (es-GDNF) test * p < 0,05, n = 30.

The analysis of this genetic expression's influence on the initial stage of the differentiation of the transfected embryonic SCs-EBs formation was examined. During research, the time of the EBs formation and their number was determined. The results of these experiments (EBs calculation was done on the 3rd and 4th days after the cell seeding) showed that the es-GDNF line cells form EBs almost concurrently with the control ones; however, the number of the EBs in the es-GDNF line cells was 40—45% lower than in the es-GPF line (Fig.6). These findings point to the fact that there is a verifiable decrease in the EB number in the es-GDNF line cells when compared to the controls, which testifies to the inhibiting GDFN influence on the earlier stages of the embryonic SC differentiation stage. 

Consequently, as a result of the experiments, the embryonic mouse SCs that express the human GDNF gene fused with the GFP gene were obtained and partly characterized. The increased expression of the given gene leads to the slowing-down of the EB formation without influencing the morphology and proliferative activity of the transfected embryonic SCs. In the future, the given cells' line may be used both to investigate the way the human GDNF gene influences further stages of embryonic SCs in vitro, especially in the neuronal direction, and for in vivo experiments with the purpose of correcting several brain pathologies connected with neurodegenerative illnesses such as Parkinson disease. 

## Acknowledgements

This paper was completed with partial support from the Ministry of Education and Science of the Russian Federation (GK no. 02.512.12.2013) and the Russian Foundation for Basic Research (grant number 07-04-12258-ofi). 
